# Clinical Report on Chronic Pleurisy, Based on an Analysis of Forty-Seven Cases

**Published:** 1852-12

**Authors:** Austin Flint


### BUFFALO MEDICAL JOURNAL

AND

### MONTHLY REVIEW.

VOL. 8. DECEMBER, 1852. NO. 7.

### ORIGINAL COMMUNICATIONS.

ART. I.—*Clinical Report on Chronic Pleurisy, based on an analysis of Forty-seven cases.* By Austin Flint, M. D.

(Continued from page 253.)

### DIAGNOSIS.

It is evident that, so far as its symptomatology is concerned, exclusive of physical signs, Chronic Pleurisy is often remarkably latent in its character, as well as insidious in its development. Symptoms pointing to pulmonary disease, rarely prominent, are frequently quite insignificant, or altogether absent; and when present even in a degree to direct attention to the chest as the seat of the malady, they afford no criteria to indicate the particular structure involved, and the nature of the affection. The rational or vital phenomena, in a large proportion of cases, are insufficient for a positive diagnosis; and, inasmuch as physical exploration is still neglected by a large class of practitioners, the disease, in proportion to its frequency of occurrence, is, perhaps, as often overlooked and confounded with other affections as any in the nosology. The cases that I have coll cted furnish proof of the correctness of this remark. In a pretty large number of these cases the histories show errors of diagnosis on the part of the physicians under whose notice they had fallen, and I am by no means assured that pains were taken in noting the history of each case to ascertain and record the facts with respect to this point. The propriety of embracing the facts pertaining to this subject in the present Clinical Report, would be more than doubtful, if they were not calculated to subserve an useful practical purpose. But inasmuch as they tend to enforce the importance of physical signs, it would be as improper to withhold them, as it would be to give them publicity simply as a matter of curiosity.

The number of cases in which errors of diagnosis are noted to have occurred, is eighteen. Of these eighteen cases, in four the disease had been treated as latent intermittent fever. This error was based, in two instances, on the fact that chills were pretty prominent, and in the other cases on the fact that the patients had been exposed to miasmata. In four cases the disease was supposed to be phthisis. In two cases the patients were thought to be laboring under continued fever, and in one case the disease was developed in the course of fever and overlooked. In the remaining seven cases, the supposed affection was in each case different. The following is a list of the diagnostic views that had been entertained: Disease of the heart, abscess between the pleura and walls of chest, bilious fever, hepatization of lung, liver complaint, general debility, and some pulmonary affection the nature of which was confessedly not known.

The diagnosis of Chronic Pleurisy, with the aid of physical exploration, is as simple and sure, as it is difficult and doubtful when the sole dependence is on the symptoms. Flatness on percussion extending from the bottom of the chest, upward, over the whole, or a considerable portion of one side, anteriorly, posteriorly, and laterally, and absence of respiratory sound, would alone lead to a correct diagnosis, with some very rare exceptions. A tumor filling the chest on one side, in part, or entirely, constitutes almost the only morbid condition in which the above combination of signs is presented, exclusive of liquid effusion. Solidified lung rarely, if ever, occasions the same absolute loss of sonoriety, in the same situation, and to the same extent, coupled with absence of all respiratory sound; and, on the other hand, the presence of a bronchial respiration in cases of large liquid effusion is an anomaly of which but a few instances are on record. But, aside from the above combination, if the liquid effusion be large, as it generally is in Chronic Pleurisy, we have the enlargement of the chest on the affected side, its comparative immobility, the widening and elevation of the intercostal spaces, the displacement of the heart affected in the left side, the contraction of the affected side after the liquid effusion has been removed wholly, or in part, by absorption, the change of level of the fluid if the quantity be moderate and the pleural surfaces free, a friction sound in some cases, the absence of the vocal fremitus frequently appreciable on the sound side, and occasionally the œgophonous modification of bronchophony. With the assistance of more or less of these collateral signs, the discrimination is easy and certain. Reference is now made to Chronic *general* Pleurisy. Circumscribed pleurisy may involve difficulties in the way of diagnosis to which it will be more appropriate to refer in another connection. To treat of the diagnosis at any length, does not fall within the scope of this Report. After indulging, however, the few foregoing remarks, I may briefly allude to one or two points of practical interest which are incidentally connected with the subject.

Chronic Pleurisy and tuberculosis of the lungs occasionally coexist. This complication does not appear to be of frequent occurrence, but it is desirable to determine the coexistence in individual cases. How is this to be done? I am not prepared to answer this question by submitting conclusions deduced from an extensive series of personal observations. The few facts, however, which have been already presented, involve the practical rule to be adopted. Recollecting this law of tuberculosis, viz., that it invades the lungs successively, on both sides, after an interval of greater or less length, we are to seek on the side which is sound as respects the pleurisy, for the various physical indications of tubercle, such as crackling, jerking respiration, sibilant or mucous râles, bronchophony, etc. If any of these signs are found to be persistent, and confined to the apex of the lung on the nonpleuritic side, they show, as under other circumstances, the presence of a tuberculous deposit on that side. If the deposit be quite abundant, and especially if it have advanced through the changes of softening and excavation, the physical indications will of course be still plainer and more unequivocal. The symptoms, as well as signs, are to be considered in resolving the question under consideration. Hœmoptysis must be regarded as a very significant event. A prominent and persisting cough, more especially if accompanied with a progressively increasing expectoration, is also ominous, since these do not ordinarily belong to the natural history of simple pleurisy. Marked emaciation, and hectic paroxysms should also lead to strong suspicions of tubercle.

Phthisis, if it do not coexist, may be developed subsequently to Chronic Pleurisy. As a sequel it is probably far less frequent in its occurrence than the views of some late writers would lead us to suppose. Of fifty-three of the cases analyzed by Dr. Blakiston, in which the patients were under observation for some years after the attack of pleurisy, not one became phthisical. So far as my observations go, they show a very small proportion of instances in which this result has followed. That it does occasionally follow, however, is not to be doubted, and the permanent changes in the chest due to the pre-existing pleurisy, offer some obstacles in the way of the physical diagnosis. It is well known to all who are practically conversant with physical exploration, that some of the signs of early tuberculosis are based on the correspondence of the two sides of the chest in a state of health. This natural harmony, which is sufficiently so for practical purposes, is permanently impaired, to a greater or less extent, by Chronic Pleurisy, as will be seen in the division of this Report to be presently taken up. The disparity which succeeds the disease, relates more especially to the resonance on percussion, the mobility, and the comparative development of the vesicular respiration. We cannot determine morbid signs involving the points just mentioned, by a comparison of the two sides of the chest, as we should do had the patient not been affected with Chronic Pleurisy. Under these circumstanc s eli n :e in fo:ming a diagnosis must measurably be placed on the same combination of signs and symptoms which have just been referred to in connection with coexisting pleurisy and tuberculosis.

With reference both to coexisting and consecutive phthisis, the study of, and practical acquaintance with all the various indications, physical and rational, of incipient tuberculosis, are eminently important to the physician. It is hardly necessary to add, that the chief object in alluding to the points just noticed, is to show how the disease which is the subject of this Report, affects the importance of these indications.

Another point of practical interest connected with the diagnosis of Chronic Pleurisy, pertains to the conversion of the liquid contents of the chest into pus; in other words, the development of empyema. How is this to be ascertained in individual cases? The data afforded by the present collection of observations, bearing on this point, will be found to be insufficient for many practical deductions; but I may anticipate so far as to say that the persistent evidences of a large accumulation of liquid effusion within the chest, showing little or no efforts at removal by absorption, and the presence of symptoms denoting no progress toward recovery, but a tendency to a fatal issue, should occasion suspicions of a purulent change. This language is comprehensive, and rather indefinite, but to determine the special significance of particular symptoms, it would be necessary to analyze a series of cases of empyema, and compare the results with those attained by the analysis of cases of ordinary Chronic Pleurisy. The materials for the former labor are not at hand. The nature of the liquid contents of the chest, it may be remarked, can be demonstrated, in individual cases, in which information on this point is desirable, by means of the exploring canula, which the recent experiments of Dr. H. I. Bowditch, of Boston, Mass., have shown may be resorted to with entire impunity, to say the least, for the purpose just mentioned. The withdrawal of the fluid by means of the canula, is another matter which relates to the subject of treatment.

### FATALITY.

On examining the fatal cases in this collection, I find that they admit of being divided into several groups, as follows: *First*, cases of death from uncomplicated pleurisy; *second*, cases in which the pleurisy was associated with other grave affections; *third*, cases in which death was due to an intercurrent disease, and, *fourth*, cases in which a fatal termination occurred from causes acting shortly after recovery from the pleurisy. I will state the facts pertaining to the fatality under these several heads.

1. *Death from uncomplicated pleurisy.* The fatality which belongs solely and directly to Chronic Pleurisy, is to be determined by facts falling under this head. Excluding cases in which the issue was unknown, together with a few cases in progress at the present time, and the number of recoveries is *nineteen*. The number of deaths from uncomplicated pleurisy was *four*. Of these four cases the fact of the disease not having been complicated, was autopsically demonstrated in two. The fact was predicated on the history and symptoms in the remaining two cases. Thus, in four of twenty-three cases of Chronic Pleurisy, a fatal result was due, so far as could be ascertained, to the pleurisy exclusively.

2. *Death from pleurisy complicated with other grave affections.* Seven fatal cases are embraced in this category. Adding these to the cases which recovered, nineteen, and the ratio of fatality is as seven to twenty-six. The affections complicating the pleurisy were in three cases tubercle; in two cases fever; in one case laryngitis leading to œdema glottidis and sudden death, and in one case pericarditis, ascites and double pleurisy.

3. *Death from an intercurrent affection.* One patient, a child, died with a bowel complaint, which occurred while the pleurisy was progressing favorably.

4. *Death from subsequent affections.* One patient died shortly after recovering from the pleurisy, with what is said to be *bilious colic.* Another patient died of a repetition of the pleurisy occurring several months after the recovery from the first attack which seemed to be complete. This case, judged by the symptoms, was complicated with pericarditis. The fact of the fatal termination in this case was never positively ascertained, but when last seen the patient was near the close of life, and it is therefore assumed that it ended fatally.

In the foregoing account are excluded four cases of perforation, all of which ended fatally, and two cases of circumscribed pleurisy one of which was fatal.

Adding together the fatal cases in all the above groups, including the four cases in which there was perforation, and the fatal case in which the pleurisy was circumscribed, and the sum is nineteen. In the same number of cases, nineteen, the patients were known to have recovered from the pleurisy, and in nine cases the termination is unknown.

From the results which have been submitted the general conclusions to be drawn are, that Chronic Pleurisy, *per se*, is fatal in only a very small proportion of cases; that the majority of deaths occurring during the progress of, or shortly after the disease, are due to its complications, or to intercurrent and subsequent affections; but taking into view all the circumstances connected with the origin, progress, and immediate tendencies of the disease, the rate of mortality is about fifty per cent.

DURATION.

*Fatal Cases.*

| No. | | *Remarks.* |
|---|---|---|
| 1. | Two months after admission into hospital. | Associated with tubercle. Autopsy. |
| 2. | Six weeks after coming under observation. Previous duration not ascertained. | Empyema. Uncomplicated. Autopsy. |
| 3. | Several weeks after coming under observation. Previous duration not ascertained. | Uncomplicated. Autopsy. |
| 4. | Seven days after admission into hospital. | Lesions of typhoid fever. Had a short time before had scarlatina. Autopsy. |
| 5. | Seven weeks after date of attack. | Supposed to be uncomplicated. No autopsy. |

| | | |
|---|---|---|
| 6. | Five months. | Supposed to be uncomplicated. No autopsy. |
| 7. | Forty days. | Patient supposed to have fever. Empyema. Autopsy. |
| 8. | Not ascertained. | Complicated with laryngitis, and sudden death from œdema glottidis. No tubercle. Autopsy. |
| 9. | Two months. | Associated with tubercle. Autopsy. |
| 10. | Not ascertained. | Complicated with ascites, pericarditis and double pleurisy. |
| 11. | Ten months. | Associated with tubercle. No autopsy. |
| 12. | Six months. | Died of what was called bilious colic. No autopsy. |
| 13. | Several months. | Died with bowel complaint while apparently recovering from pleurisy. No autopsy. |
| 14. | About two years. | Died from second attack after having appeared free from the disease for a year. No autopsy. |

It is evident that Chronic Pleurisy, when it proves fatal, has no definite duration. When it is complicated with other affections, the issue, of course in a greater or less degree, is dependent on the nature and extent of the latter. When uncomplicated, however, so far as any conclusion on this point can be drawn from the foregoing data, the duration is variable.

### *Cases not Fatal.*

In order to determine with precision the duration in cases ending in recovery, it is necessary to fix, in each case, the period when the processes of restoration are completed. These processes involve absorption of the liquid contained in the pleural cavity, the organization of the effused fibrin, etc. It must be difficult, with all the aid which physical exploration affords, to arrive at exact information on this point. An analysis of a collection of observations relating thereto would be interesting. The histories that I have preserved do not embrace data sufficient for such an analysis. As before stated, in a large proportion of the cases the patients did not remain under my observation during the progress of the disease; and in those in which the opportunity was offered, pains were not taken to obtain and record the results of repeated examinations of the chest, while convalescence was going on. I have uniformly observed that at the time when, judged by rational signs, recovery was nearly, or quite established, the physical evidences of the presence of more or less liquid in the chest, persisted. In several instances the patients regarded themselves well, and were able to take active exercise, while the quantity of liquid effusion was considerable. I have already cited illustrations of this, and also of the fact that active occupation may be continued from the beginning of the disease during the greater part or the whole of its career. Even many months after apparent recovery, I have found flatness and absence of respiration over the lower third of the affected side. For the completion of the processes of restoration a long period is required. I regret that my recorded observations do not enable me to state the average period with more exactness.

There is another aspect under which the duration may be viewed, viz., from the date of attack to the time when patients are sufficiently recovered to resume their usual avocations. The nature of the occupation, and other circumstances, irrespective of the condition of the chest, it is obvious, will affect the duration in this aspect. I will, however, give the facts in the few instances in which the histories contain information relative to this point.

Case 1. The patient was a soldier, and remained in hospital thirty-two days, when he was considered by the medical officer, and himself, well enough to be returned for duty. The chest, however, was two-thirds filled with liquid.

Case 2. Recovered sufficiently to resume the duties of a physician in about three months. For several months afterward he was feeble, and subject to cough. Recovered at length his former health.

Case 3. Was able in about four months, to engage in tolerably active business, but was short-winded on exertion, and the physical signs of considerable liquid effusion continued. He progressively improved, until he recovered perfect health.

Case 4. Apparently quite recovered in about six months.

Case 5. After a few months (precise number not stated) able to be up and about, but not able to engage in active business for three years after date of attack. During all this time physical evidences of liquid effusion continued.

Case 6. Patient considered himself quite well in about four months.

Case 7. Entered hospital a week after attack, and was discharged convalescent in two months. Condition of chest at time of discharge not noted.

Case 8. Able to be up and walk about freely in about six months. A year afterward reported quite well, and able to do housework, being a female domestic. The quantity of liquid effusion in this case was very large.

It appears from these cases that in Chronic Pleurisy, recovery sufficiently to resume the active duties of life, is not likely to occur within a very short period. Several months, it may be expected, will elapse before the patient will reach this stage of improvement; and complete restoration will require an indefinite period beyond apparent convalescence.

### REMOTE EFFECTS.

What remote effects, local and general, follow Chronic Pleurisy? Is the chest apt to be permanently damaged? To what extent, if at all, are the physical powers of the organism usually impaired? Is there a liability to renewed attacks, or is the patient peculiarly exposed to the supervention of other affections? These questions, it is obvious, are to be answered by reference to the results of an analysis of a sufficient number of cases remaining under observation for a considerable length of time after recovery from the disease. The group of cases in this collection in which the diagnosis was retrospective, will furnish some facts pertaining to this subject. In a portion, also, of the cases belonging to the other groups, the disease occurred several years ago, the condition of the patients in the mean time being known. Several of the cases, however, are of too recent date to be studied in this point of view; and in several the patients were lost sight of directly, or shortly after coming under notice. In fourteen of the forty-seven cases the consecutive history extends over a sufficient period to furnish information relating to remote effects. The facts noted with respect to these cases, respectively, are as follows:

Case 1. In this case the patient had experienced an attack of Chronic

Pleurisy forty years before her death in April, 1847. She was confined to her room with the pleuritic affection for several months, and remained an invalid for several years. The history with greater particularity of detail was not obtained. After recovering entirely from the disease, she enjoyed excellent health until a few years before her death, when she suffered from palpitations and paroxysms of sinking. She had no medical treatment for these symptoms until about a fortnight before her death, when she came under the care of Dr. Wilcox, of this city. I saw her in consultation with Dr. W. She presented the physical evidences of greatly enlarged heart, without valvular lesions. The action of the heart was exceedingly irregular, and accompanied by paroxysms of great suffering from orthopnœa. At the autopsy the heart was found to weigh 16 oz., the cavities greatly dilated, the walls not thickened, and the valves free from disease.

In the right chest, which had been the seat of the pleurisy forty years before, the pleural surfaces were united throughout, with firm adhesions. The pleural cavity was entirely abolished. Lungs on both sides free from disease. Moderate effusion of serum had taken place in the left pleural cavity.

Case 2. This patient experienced the disease five years ago. After several months he recovered sufficiently to resume the duties of a medical practitioner, but he remained feeble, and was subject to cough for about a year. He has since continued in good health, and is now free from any evidences of pulmonary or other disease.

Case 3. This patient was supposed, from the history of symptoms, to have had the disease three years prior to the time of examination. She was then, i. e., at time of examination, probably laboring under tuberculosis, and died a few months afterward.

Case 4. This case occurred a little over four years ago. After a few months the patient began gradually to resume active occupation as an outdoor clerk in a lumber yard. After the lapse of one or two years he was able to endure as much physical activity as the most robust, and has remained up to the present time in perfect health. The quantity of liquid effusion in this case was very large, occurring in the left side, and removing the heart to the right of the sternum.

Case 5. The patient was a child six or seven years of age. The case was examined one and a half years after the date of the attack. The only ailment at that time was debility, and deficient growth. Four years after the attack, it had continued well, but was more feeble, and had grown less than the average of children of the same age.

Case 6. This case came under my notice four years after an attack of Chronic Pleurisy. The patient was laboring under tuberculosis, which, from the history, appeared to have supervened about two years after the pleuritic attack. She died a few months afterward.

Case 7. This patient came to me for an examination of the chest seven months after an attack of Chronic Pleurisy. There was at this time marked contraction of the chest, with the physical evidences of liquid effusion still at the lower part of the chest. This examination was made about three years ago. The patient has been very feeble for months, and it is not expected that she will live long. She is supposed to be in the last stage of tuberculosis. She resides at a great distance, and the above information is all that I have obtained relative to the progress of the case.

Case 8. This case came under observation six months after the date of attack. The patient had then quite recovered; the liquid effusion was nearly absorbed, and the chest considerably contracted. It is now three and a half years after the attack. The patient, since the time of my examination, has been in good health, and so continues at this time.

Case 9. The patient in this case was a lad ten years of age. He came under my notice two months after the date of attack. The chest was then filled with liquid effusion. He recovered after a few months, and has remained up to the present time, a period somewhat less than three years, in excellent health.

Case 10. This case came under observation a little more than two years ago. The patient had had an attack of Chronic Pleurisy twenty months previous. He presented, still, evidences of considerable liquid effusion. The chest afterward exhibited marked contraction. Since coming under my observation he has had two attacks of hæmoptysis, in one of which the hæmorrhage was pretty profuse, and continued more or less for several days. He has, however, been steadily improving in flesh and strength, and at this time appears in perfect health, being able to engage in active occupation as an out-door clerk.

Case 11. In this case the patient was examined by me three weeks after the date of attack. The quantity of liquid effusion was then moderate. This was fourteen months ago. He recovered sufficiently to resume the duties of an in-door clerk, but has always remained rather feeble, with more or less cough. On a second examination, a year after the first, the physical signs of tuberculosis were unequivocal. There is ground for the suspicion that the tuberculous deposit preceded the attack of pleurisy. My knowledge of the case was derived at the examinations just referred to. The patient has removed to a southern climate.

Case 12. In this case I made an examination of the chest about fourteen months after the date of the attack. The patient was a farmer, 47 years of age. There was marked contraction of the chest. There were no symptoms of pulmonary disease present, the only subject of complaint being a diminished ability to perform active labor.

Case 13. The patient in this case, came under treatment four weeks after the date of attack. This was a little less than two years ago. She has remained under observation up to the present time. The left side was the side affected, and the quantity of liquid effusion was very large, removing the heart to the right of the sternum. Great contraction of the chest followed the absorption of the effused fluid. The patient, a female domestic, for a year past has been able to do laborious house work, and is now in excellent health.

Case 14. The date of the disease in this case was five years prior to the examination. The left side was still considerably contracted. There existed at the time of the examination, hypertrophy of the heart, to which the symptoms present in the case were referable. From the history it appeared probable that the pleurisy was complicated with pericarditis.

Reviewing the facts contained in the foregoing account of a limited number of cases, it appears that in precisely one-half (seven) the patients preserved for several years after recovery, so long as they remained under observation, or up to the present time, excellent health. In two other cases the health was tolerably good.

In two cases hypertrophy of the heart supervened, but in one of these cases the latter affection was discovered at a period quite remote, at an advanced age; and in the other case the pleurisy was probably complicated with pericarditis. The cases furnish no evidence of a special tendency, after Chronic Pleurisy, to any disease except tubercle. In *three* cases the subsequent development of tuberculosis was highly probable, although not demonstrated, and in one case the latter disease was positively determined. It has been a current opinion that Chronic Pleurisy predisposes strongly to phthisis. There is reason to think that this opinion is incorrect, having been based on isolated observations. In the analysis by Dr. Blakiston, to which I have before referred, not one out of fifty-three cases became phthisical during the lapse of several years after recovery from pleurisy. This result is striking, for it might be expected that out of so large a number of cases of any disease occurring as does pleurisy, for the most part, at an early age, tubercle would be likely to affect, in the course of several years, a certain proportion. In estimating the influence of pleurisy, or any disease, in determining the subsequent development of tubercle, the liability to the latter, irrespective of preceding affections, is, of course, to be taken into account. Giving to this consideration a certain degree of weight, and bearing in mind that in four of the five cases neither the presence of tubercle subsequent, nor its absence prior to the pleurisy, were demonstratively established, and it follows that the few cases in the present collection analyzed with reference to this point, show but a slight, if any tendency to phthisis to be among the consequences of Chronic Pleurisy.

The remote effects of Chronic Pleurisy upon the chest, so far as they affect the results of physical exploration, constitute an interesting branch of inquiry. For several years after recovery, the symmetry of the two sides, as respects the equality of percussion sounds, and the relative intensity of the vesicular murmur, is impaired. It is probable that this effect frequently continues to some extent through life. A series of careful examinations of the chests of persons who had experienced pleurisy many years previous is a desideratum. The object, however, relates mainly to the subject of physical diagnosis. There is one point to which no allusion was made in connection with the physical signs, which may properly be ranked among the remote effects. This is the gradual expansion of the chest, on the affected side, after contraction has taken place in consequence of absorption of the liquid effusion. I have not taken pains to make careful examinations relative to this point. I have had occasion, however, to observe that this subsequent expansion does take place in cases in which there had been marked contraction. In case No. 4, in the foregoing group, four years after the date of attack, the chest on the affected side had enlarged so as to appear nearly equal in capacity to the other. In this case the quantity of liquid effusion was very great, removing the heart to the right of the sternum, and contraction subsequent on absorption was present in a marked degree two years after the date of attack. In case No. 13, there also existed very large effusion removing the heart beyond the right margin of the sternum. Absorption was followed in this case by very marked contraction. At the present time, however, two years after the date of attack, the affected side has expanded so that on measurement on a line passing over the nipple it falls short by less than half an inch the dimensions of the right side, the relations of the two sides to each other being about normal. Considerable contraction is permanent in some instances. This was shown in case No. 14, in which it was observed five years after the date of attack. It is probable that age has an influence in determining the degree of expansion. In the case last referred to, the age of the patient is not stated, but he was in the neighborhood of forty years. In the two cases previously referred to the patients were young, both being twenty years of age.

### CIRCUMSCRIBED PLEURISY.

The pleurisy was circumscribed in two cases. In one, the character of the disease was ascertained by the symptoms and signs during life, the patient recovering; in the other, the affection was supposed to be seated in the liver, and the diagnosis rectified by the autopsy, the case proving fatal. The circumstances belonging to the history of both cases, considering the infrequency of this form of uncomplicated pleurisy, seem to warrant a brief report of each case.

Case 1. The patient was a female, fourteen years of age. The case was referred to me for examination by Dr. G. Conger, of Niagara Falls, in July, 1850. At the time of my examination the following were the facts noted: "She menstruates regularly. Aspect is not morbid. She is not emaciated, and has not lost flesh of late. She is highly nervous, weeping at the apprehension of a physical exploration. There is no spinal tenderness. Appetite and digestion tolerable. The friends state that, when an infant, she had hooping cough severely, which was followed by inflammation of the lungs. She has ever since been subject to a cough, loud and hard, and attended usually by small, transparent, frothy expectoration. Five weeks ago she suddenly expectorated a quantity of fœtid purulent matter. Since that time she has had several (seven or eight) similar attacks. She continues to expectorate the same matter for a day or two after the attack commences, and then the expectoration is no greater than usual. The cough has diminished since these discharges took place. She has kept her bed for a week during the past five weeks, and now her strength is regained so that she came to this city without much fatigue.

On examination of the chest I find moderate dullness on percussion over the upper third of the right side, with no distinct abnormal modification of the respiratory sound. Absolute flatness exists over the lower, and most of the middle third, on the right side, with absence of respiration, in front and laterally. Posteriorly, on the right side, the resonance is clear as low as is usual in a state of health. I discover no râles, nor cavernous nor bronchial respiration. There is tenderness on percussion over the right mamma. No tenderness, nor evidences of enlargement of the liver.

From the foregoing signs, and the history, I inferred the non-existence of tuberculosis; and that the purulent expectoration, occurring, thus, at several epochs, was due to circumscribed collections between the pleural surfaces, discharging through the bronchial tubes, rather than to abscesses in the substance of the lung, or in neighboring organs. I have given all the data which were noted, so that the reader may form his own opinion as to the correctness of the diagnosis.

I saw the patient again in March, 1851. She was then quite well. On a slight examination, however, I found that there was still flatness over the lower part of the chest on the right side. She was in good flesh, and had no cough. She had had no purulent expectoration for some time.

I have since been informed by Dr. Conger, that subsequent to the time of my last seeing her, she had another recurrence of purulent expectoration, from which she recovered as before, and afterward appeared in good health.

Case 2. The patient was an Irishman, a peddler and laborer, aged forty-five years. He came under my care at the Buffalo Hospital of the Sisters of Charity, at the commencement of my winter service, Oct. 1, 1851. He had entered about a fortnight before. The previous history was as follows: Attacked with cough about two years ago, and has had more or less cough from that time to the present. Has had several attacks of hæmoptysis, the first having been early in the disease. Has occasionally had chills, and stitch pains in the chest frequently. Has had diarrhœa for the last ten days, which still continues. Has to-day attempted to sit up for the first time since his admission, and for two weeks previous. Was not attended by any physician before entering the hospital.

*Present Symptoms:* Not greatly emaciated. Aspect pallid. Pulse 72, and extremely feeble. Two or three dejections daily. Expectoration copious and purulent.

Physical examination was deferred. The case was presumed to be one of tuberculosis.

Oct. 7, it was noted that the patient had expectorated during the past six hours about ten ounces of pus, and was still raising it freely at short intervals. Physical examination on this day gave the following results: Emaciation not sufficient to render the outline of the ribs visible. Respiration not labored, 24 per minute. Clear resonance at the summit of the chest on both sides; the pitch of sound being somewhat raised on the right side, but the resonance clear. Flatness on the right side extends from the bottom of the chest as high as the fourth rib. The chest on the right side, at the inferior part, *and below*, is tender to the touch. Some degree of tenderness, also, exists on the left side. He refers the pains to the inferior and lateral portions of the right side. The pain is not severe. It is lancinating.

Posteriorly, in the interscapular space, the resonance is clear on both sides, the pitch being elevated on the right side. Below the inferior angle of the scapula, and over the lower part of the scapula, on the right side, flatness exists.

Laterally, on the right side, there is flatness.

The respiration on the left side is exaggerated. Posteriorly, on the right side, the respiratory sound is feeble, tubular, and accompanied by a fine mucous or subcrepitant râle. At the upper third, on the right side, anteriorly it is feeble but not tubular. This obtains above the fourth rib. Below the fourth rib there is absence of respiratory sound, and a distinct, but not loud friction sound heard in both acts of respiration.

Oct. 12. Quantity of expectoration diminished by one-half, or two-thirds according to a rough estimation. Diarrhœa continues to be a troublesome symptom, and the house student thought he had discovered pus in the evacuations. The matter expectorated is pure pus, with little or no mucus.

In view of the foregoing facts, hepatic abscess, evacuating through the lungs, was suspected. The reasons for this opinion are set forth in the following quotation from the hospital records: "From the symptoms and signs in this case, the probable diagnosis is, purulent formation within the liver, evacuating through the bronchi. This presumptive diagnosis is based on the following points:

"1. The profuse purulent expectoration without the physical signs of a cavity in the lungs.

"2. The slight evidences of disease at the summit; consisting only of slight elevation of pitch on percussion on the right side, the left side appearing perfectly healthy; the respirations not accelerated nor labored; the pulse unaffected; the emaciation not extreme, etc.—all these circumstances excluding tuberculosis.

"3. Empyema being excluded by the absence of the evidences of considerable pleuritic effusion, i. e., no signs that the right chest *has been* distended by liquid effusion, nor that it now exists in much quantity. In this connection it should be stated that distinct bronchophony exists at the angle of the scapula on the right side.

"4. Pneumonitis is excluded by the absence of evidence in the history that this disease has existed; by the improbability of this disease, had it existed, eventuating in abscess; and the absence of the physical signs of a cavity.

"5. The presence of tenderness below the chest on the right, over the site of the liver.

"6. Pus in the evacuations, if this be true. The presence of this symptom needs confirmation.

"7. All the signs and symptoms are rationally explicable on the supposition of a purulent formation in the liver, at the same time that other supposable affections adequate to the production of so large a quantity of purulent expectoration must be excluded."

It will be perceived that circumscribed Chronic Pleurisy was overlooked in canvassing the evidence in behalf of the several affections which might be supposed to be present in this case. The patient died on the first day of the November following, and the autopsy revealed the following condition of the chest:

At the inferior part of the right chest there existed a pleuritic abscess about five or six inches in width, extending from the lower part of the sternum quite around the right side of the chest. The pleural surface in the strip just mentioned, exhibited a rough granular appearance, and the membrane was much thickened. Above the strip the pleural surfaces were united by rather feeble adhesions. The lower lobe of the lung on the right side was solidified. The lungs otherwise healthy. The situation of the pleuritic abscess, or circumscribed empyema, was such as to coincide with the physical signs.

A circumstance mentioned by the patient, although not recorded in the previous history, may, perhaps, serve to explain the production of the circumscribed pleurisy in this case. For some time before being attacked with the disease, he had followed the avocation of a pedler, and had been accustomed to carry a pack, resting heavily on the right side, and pressing particularly on the lower part of the chest on that side. He stated that it frequently occasioned severe pain in the situation in which he experienced pain during the progress of the disease, and over the site of the pleurisy. The patient referred the origin of the disease to this cause.

(To be continued.)

